# Influencing factors of patient delay for decision-making in older patients with acute coronary syndrome: a study based on the Andersen’s Behavioral Model

**DOI:** 10.3389/fpubh.2026.1788401

**Published:** 2026-03-20

**Authors:** Manjun Wang, Xiaoyan Wu, Yuting Ke, Chenping Zhu, Jun Xie, Jinhua Jin

**Affiliations:** Nursing Department, Sir Run Run Shaw Hospital, Zhejiang University School of Medicine, Hangzhou, Zhejiang, China

**Keywords:** acute coronary syndrome, Andersen’s Behavioral Model, cross-sectional study, older adults, patient delay

## Abstract

**Objective:**

This cross-sectional study aimed to identify factors influencing patient delay in older adults with acute coronary syndrome (ACS) using Andersen’s Behavioral Model at a tertiary hospital in Zhejiang Province, China.

**Methods:**

A total of 153 older adults diagnosed with ACS who survived to hospital admission were included from January 2023 to June 2023. Logistic regression analysis was used to determine factors independently associated with a longer patient delay following symptom onset.

**Results:**

The median delay in seeking medical care was 100.00 min (55.00, 450.00 min). Female sex (odds ratio [OR] = 2.762, 95% CI: 1.067–7.153), advanced age (≥75 years; OR = 4.244, 95% CI: 1.519–11.859), diagnosis of unstable angina (OR = 3.430, 95% CI: 1.281–9.182), and history of diabetes (OR = 3.921, 95% CI: 1.364–11.273) were significant factors associated with prolonged patient delay. In contrast, attributing symptoms to cardiac disease (OR = 0.269, 95% CI: 0.088–0.823), engaging in consultation-seeking behavior following symptom onset (OR = 0.326, 95% CI: 0.124–0.856), and a history of heart disease (OR = 0.274, 95% CI: 0.095–0.790) were associated with reduced likelihood of delay.

**Conclusion:**

To reduce patient delay among older adults with ACS, it is important to improve their ability to recognize ACS symptoms, especially in female, older, and patients with diabetes, and those with cardiovascular risk factors but no prior cardiac diagnosis. Accessible consultations could support older patients in making quick decisions about care-seeking behavior.

## 1 Introduction

Acute coronary syndrome (ACS) is a group of clinical syndromes based on the pathological foundation of atherosclerotic plaque rupture or erosion, followed by the formation of complete or incomplete occlusive thrombi (1). It primarily includes ST-segment elevation myocardial infarction (STEMI), non-ST-segment elevation myocardial infarction (NSTEMI), and unstable angina (UA), with the first two collectively referred to as acute myocardial infarction (AMI). As a common acute cardiovascular disease, ACS is characterized by rapid onset, rapid changes, and poor prognosis, posing a significant threat to human health and survival. According to the American Heart Association, approximately 1,266,000 Americans are discharged with an ACS diagnosis annually (2). In China, the incidence rate of AMI was 79.7 per 100,000, and its mortality rate increased annually, reaching 63.25 per 100,000 in urban areas and 83.26 per 100,000 in rural areas in 2021 (3). Adults aged ≥75 years are disproportionately affected, accounting for 30–40% of all hospitalizations for ACS, with mortality predominantly occurring in this population (4). Therefore, ACS imposes a substantial burden on society, particularly on the older population.

Reperfusion therapy is currently the most effective treatment for ACS. However, the efficacy of reperfusion therapy is closely related to the time elapsed since symptom onset. The longer the delay between symptom onset and reperfusion, the worse the prognosis. Studies have shown that reperfusion therapy within 1 h of symptom onset is optimal, as it can reduce complications and improve survival rates (5). For every 30-min delay in reperfusion therapy, the 1-year mortality risk increases by 7.5% (6). Although early treatment is crucial for ACS, significant delay in seeking medical care persists among patients with ACS, with 73.96–81.92% of Chinese patients with AMI dying before reaching the hospital (7). Therefore, a delay in seeking medical care warrants urgent attention.

Patient delay, defined as the time taken by the patient from symptom onset to the decision to seek treatment, is the most significant factor affecting the time to reperfusion therapy (8, 9). Reports indicate that only 54% of patients seek medical help within 2 h of chest pain onset, while 24% seek medical help more than 12 h after the symptom onset (10). Several studies have shown that age is a key factor influencing patient delay among patients with ACS. Delay time for older patients is significantly longer than that for younger patients (11–13), which may lead to poorer outcomes (13). Therefore, healthcare professionals must identify the factors contributing to delays in older patients with ACS and develop strategies to reduce them.

The Andersen model, also known as the Behavioral Model of Health Service Use, has become the mainstream model for understanding healthcare utilization. It comprises predisposing, enabling, and need factors, providing a comprehensive framework that explains how these factors influence an individual’s use of healthcare services (14). Although the Andersen’s Behavioral Model has been extensively applied in healthcare research, no studies have investigated its use in examining factors influencing patient delay among older patients with ACS.

This study aimed to systematically investigate the determinants of patient delay in older patients with ACS using Andersen’s Behavioral Model. These findings are expected to provide an empirical foundation for developing targeted intervention strategies to reduce patient delays and improve clinical outcomes in this vulnerable population.

## 2 Materials and methods

### 2.1 Study design and participants

This study was developed following the STROBE guidelines (see checklist in Supplementary material). A hospital-based cross-sectional study was conducted between January 2023 and June 2023 at a tertiary hospital. All older patients diagnosed with ACS who survived to hospital admission during the study period were enrolled. The inclusion criteria were as follows: (1) a confirmed diagnosis of ACS, including STEMI, NSTEMI, or UA; (2) age ≥60 years (according to the World Health Organization, older adults are typically defined as individuals aged 60 years and above); (3) the ability to accurately recall symptom onset time, decision-making time for seeking medical care, and events preceding hospital admission; and (4) being conscious, oriented, and possessing adequate communication skills. The exclusion criteria were as follows: (1) individuals with psychiatric disorders and (2) those with other severe comorbidities that may impair symptom awareness (e.g., advanced cancer, stroke, end-stage renal disease, or other debilitating conditions).

### 2.2 Definition of patient delay

In this study, patient delay was defined as the interval between symptom onset and the decision to seek medical care, including contacting emergency medical services or independently deciding to visit a healthcare facility. A decision time exceeding 120 min is classified as a delay in seeking medical care.

### 2.3 Conceptual framework/variables

According to Andersen’s Behavioral Model, potential predictor variables are categorized into three domains: predisposing, enabling, and need factors (14). Based on this framework, the variables collected in this study were mapped to each domain as follows:

(i) Predisposing factors are individual characteristics that exist prior to illness, including demographics, social structure, and health beliefs. In this study, demographic characteristics included sex, age, and educational level (illiteracy and primary education/up to SSC and above SSC). Genetic characteristics were assessed based on family history of heart disease (present/absent). Social structural characteristics included marital status (married/no spouse or partner), cohabitation status (living alone/cohabiting), and residence (urban/rural). Health beliefs and behaviors included contracted family physician (present/absent), smoking status (current smoker/former smoker/non-smoker), medication adherence (no medication needed/adherent to medication/non-adherent to medication), and frequency of health examinations (none/occasional/regular).

(ii) Enabling factors are resources or conditions that facilitate or impede health service use. In this study, enabling factors included annual household income per capita (average or above/below average); type of medical insurance (provincial-level/municipal-level/rural residents’ basic medical insurance), which affected reimbursement rates; place of symptom onset (home/public place); travel time from symptom onset location to the nearest hospital; level of the nearest hospital to the location of symptom onset (tertiary/non-tertiary); and presence of bystanders at the time of symptom onset (presence/absence).

(iii) Need factors represent perceived or evaluated health status and need for care. In this study, need factors included diagnosis (AMI/UA), perceived disease severity (mild/moderate/severe), attribution of current symptoms to cardiac disease (yes/no), consultation-seeking behavior following symptom onset (whether the patient consulted with family members, friends or contracted family physician about symptom management or the need to seek medical care after symptom onset), documented history of heart disease (yes/no), history of percutaneous coronary intervention (PCI) or coronary artery bypass grafting (CABG), and history of other chronic diseases (hypertension, diabetes, stroke, chronic obstructive pulmonary disease, malignancy, and chronic kidney disease).

A visual summary of the mapping between collected variables and Andersen’s domains is provided in Table 1.

**Table 1:** Mapping of collected variables to Andersen’s Behavioral Model domains.

Predisposing factors	Enabling factors	Need factors
Sex Age Educational level Family history of heart disease Marital status Cohabitation family physician Smoking status Medication adherence Frequency of health examinations	Income Type of medical insurance Place of symptom onset Travel time to the nearest hospital Level of the nearest hospital Presence of bystanders	Diagnosis Perceived disease severity Attributing symptoms to cardiac disease Consultation seeking behavior History of heart disease History of PCI or CABG History of diabetes History of other chronic diseases

### 2.4 Data collection

#### 2.4.1 Patient delay time

Data on patient delay times were collected within 24 h of hospital admission to minimize recall bias. Symptom onset was defined as the time when the patient first perceived relevant symptoms. The time of seeking medical care was defined as the time at which emergency services were contacted or when the patient independently departed for a healthcare facility. Time records were documented as precisely as possible, with a target accuracy of 15-min intervals. For example, if a patient reported symptom onset between 14:00 and 14:30 and contacted emergency services at approximately 15:00, the symptom onset time was recorded as 14:15 and the time of seeking medical care as 15:00. In this case, the patient delay time would be calculated as 45 min. To minimize recall bias, symptom onset times were corroborated with family members or bystanders whenever possible and cross-checked against emergency medical service records.

#### 2.4.2 Variables

All variables other than patient delay time were collected through structured patient interviews conducted within 24 h of admission, supplemented by review of medical records.

### 2.5 Statistical analysis

Data analysis was performed using the Statistical Package for the Social Sciences (SPSS) version 26.0 (IBM). Continuous variables are reported as means and standard deviations, or medians and interquartile ranges (depending on whether the data are normally distributed). Categorical variables are presented as frequency and percentage. Continuous variables (age and travel time from the symptom-onset location to the nearest hospital) were dichotomized for univariate analysis. Age was dichotomized at 75 years based on previous literature indicating a higher risk in this subgroup. Travel time was dichotomized based on the median value to facilitate clinical interpretation for emergency service planning. The chi-square (*χ*^2^) test or Fisher’s exact test was used for univariate analysis. Variables with *p*-values <0.05 in the univariate analysis were selected for inclusion in the logistic regression analysis. Sample size was determined based on the events-per-variable (EPV) principle for logistic regression. With 67 patients experiencing the outcome (decision delay >120 min), an EPV of at least 10 would support the inclusion of up to 6–7 predictors in the multivariable model. To avoid overfitting and select the most parsimonious set of predictors, we employed a backward stepwise elimination procedure based on the likelihood ratio test. All variables with *p* < 0.05 in the univariate analysis were entered into the initial multivariable model. Variables were sequentially removed if their *p*-value exceeded 0.05 in the likelihood ratio test, and the procedure continued until all remaining variables had *p* < 0.05. To assess multicollinearity among the independent variables included in the multivariable logistic regression, we calculated the variance inflation factor (VIF) for each variable using linear regression analysis. VIF values greater than 10 were considered indicative of significant multicollinearity. A *p*-value < 0.05 was considered significant in the multivariate analysis.

### 2.6 Ethical statement

This study was approved by the appropriate institutional review board and ethics committee (reference number: 20200716-165). All experiments were performed in accordance with the relevant guidelines and regulations (e.g., the Declaration of Helsinki). Written informed consent was obtained from all participants.

## 3 Results

### 3.1 Participant characteristics

The study investigated 153 older patients diagnosed with ACS, of whom 84 (54.9%) had AMI, and 69 (45.1%) had UA. The mean age of the participants was 73.05 ± 7.00 years (range: 60–91 years), with 66.7% being males. Among the participants, 81.0% were married, approximately two-thirds (68.6%) lived in urban areas, and 17.6% lived alone. The demographic characteristics of the patients are presented in Table 2.

**Table 2:** Demographic characteristics of the study patients (*n* = 153).

Characteristics		Number	Frequency
Age	60–74 years	85	55.6
	≥75 years	68	44.4
Sex	Male	102	66.7
	Female	51	33.3
Educational level	Illiteracy and primary education	102	66.7
	Up to SSC and above SSC	51	33.3
Family history of heart disease	Absent	117	76.5
	Present	36	23.5
Marital status	Married	124	81.0
	No spouse or partner	29	19.0
Cohabitation status	Cohabitating	126	82.4
	Living alone	27	17.6
Residence	Urban	105	68.6
	Rural	48	31.4
Annual household income per capita	Below average	78	51.0
	Average or above	75	49.0
Type of medical insurance	Provincial-level	40	26.1
	Municipal-level	86	56.2
	Rural residents basic medical insurance	27	17.7

SSC, secondary school certificate; ACS, acute coronary syndrome; AMI, acute myocardial infarction; UA, unstable angina; PCI, percutaneous coronary intervention; CABG, coronary artery bypass grafting; COPD, chronic obstructive pulmonary disease; No spouse or partner includes patients who were widowed, divorced, or never married.

### 3.2 Distribution of patient delay

Among the 153 participants, 67 (43.8%) sought medical care more than 120 min after symptom onset, and the remaining 86 (56.2%) sought medical care within 120 min of symptom onset. Specifically, 35.7% (30/84) of patients with AMI experienced a delay exceeding 120 min, compared with 53.6% (37/69) of patients with UA. The median patient delay was 100 min (IQR: 55.0–450.0 min), with 80 min (IQR: 55.0–315.0 min) for those with AMI and 220 min (IQR: 60.0–620.0 min) for those with UA. The results are summarized in Table 3.

**Table 3:** Total delay time (min) in older patients with ACS.

Diagnosis	Range	Mean	Median (P25, P75)
ACS	15.00–2260.00	281.80	100.00 (55.00, 450.00)
AMI	15.00–2260.00	233.51	80.00 (55.00, 315.00)
UA	20.00–1320.00	340.58	220.00 (60.00, 620.00)

ACS, acute coronary syndrome; AMI, acute myocardial infarction; UA, unstable angina.

### 3.3 Univariate analysis results

Twenty-nine variables were analyzed to identify the factors contributing to patient delay in patients with ACS (Table 4). The results showed that sex, age, cohabitation status, annual household income per capita, diagnosis, perceived disease severity, symptoms attributed to cardiac disease, consultation-seeking behavior, history of heart disease, history of PCI or CABG, and history of diabetes were associated with patient delay (*p* < 0.05). These 11 variables were entered into the initial multivariable logistic regression model.

**Table 4:** Univariate analysis of factors influencing patient delay in older patients with ACS.

Variables	Categories	Patient delay		OR (95% CI)	*p*
		≤120 min (*n* = 86)	>120 min (*n* = 67)		
Sex	Male	65	37	2.510 (1.261–4.996)	0.008
	Female	21	30		
Age	60–74 years	55	30	2.188 (1.140–4.202)	<0.001
	≥75 years	31	37		
Educational level	Illiteracy and primary education	62	40	1.744 (0.885–3.437)	0.107
	Up to SSC and above SSC	24	27		
Family history of heart disease	Absent	66	51	1.035 (0.488–2.196)	0.928
	Present	20	16		
Marital status	Married	72	52	1.484 (0.659–3.338)	0.339
	No spouse or partner	14	15		
Cohabitation status	Cohabiting	77	49	3.143 (1.308–7.552)	0.008
	Living alone	9	18		
Residence	Urban	63	42	1.301 (0.842–2.012)	0.162
	Rural	23	25		
Contracted family physician	Present	25	25	0.689 (0.349–1.358)	0.281
	Absent	61	42		
Smoking status	Current smoker	42	34	1.080 (0.752–1.552)	0.260
	Former smoker	20	9		
	Non-smoker	24	24		
Medication adherence	No medication needed	29	14	1.770 (0.904–3.464)	0.215
	Adherent to medication	55	51		
	Non-adherent to medication	2	2		
Frequency of health examinations	None	18	14	0.849 (0.558–1.292)	0.419
	Occasional	29	29		
	Regular	39	24		
Annual household income per capita	Below average	37	41	0.479 (0.250–0.918)	0.026
	Average or above	49	26		
Type of medical insurance	Provincial-level	23	17	1.110 (0.682–1.807)	0.880
	Municipal-level	49	37		
	Rural residents’ basic medical insurance	14	13		
Place of symptom onset	Home	58	52	0.598 (0.288–1.240)	0.165
	Public place	28	15		
Travel time to the nearest hospital	≤25 min	44	38	0.799 (0.421–1.519)	0.494
	>25 min	42	29		
Level of the nearest hospital	Non-tertiary	25	20	0.963 (0.478–1.940)	0.916
	Tertiary	61	47		
Presence of bystanders	Absence	21	22	0.661 (0.325–1.342)	0.251
	Presence	65	45		
Diagnosis	AMI	54	30	2.081 (1.086–3.988)	0.026
	UA	32	37		
Perceived disease severity	Mild	9	27	0.368 (0.228–0.594)	<0.001
	Moderate	40	26		
	Severe	37	14		
Attribute symptoms to cardiac disease	No	38	55	0.173 (0.081–0.368)	<0.001
	Yes	48	12		
Consultation-seeking behavior	No	32	41	0.376 (0.195–0.725)	0.003
	Yes	54	26		
History of heart disease	No	12	24	0.291 (0.132–0.639)	0.002
	Yes	74	43		
History of PCI or CABG	No	66	66	0.050 (0.007–0.383)	<0.001
	Yes	20	1		
History of hypertension	No	31	27	0.835 (0.433–1.611)	0.591
	Yes	55	40		
History of diabetes	No	64	39	2.089 (1.052–4.147)	0.034
	Yes	22	28		
History of stroke	No	77	64	0.401 (0.104–1.544)	0.172
	Yes	9	3		
History of COPD	No	76	55	1.658 (0.669–4.112)	0.272
	Yes	10	12		
History of malignancy	No	80	61	1.311 (0.403–4.266)	0.652
	Yes	6	6		
History of chronic kidney disease	No	81	61	1.593 (0.465–5.465)	0.456
	Yes	5	6		

SSC, secondary school certificate; ACS, acute coronary syndrome; AMI, acute myocardial infarction; UA, unstable angina; PCI, percutaneous coronary intervention; CABG, coronary artery bypass grafting; COPD, chronic obstructive pulmonary disease.

### 3.4 Multivariable logistic regression results

After backward stepwise elimination based on the likelihood ratio test (removal criterion *p* > 0.05), the final model retained 7 variables (the coding scheme for independent variables is detailed in Table 5, and the outcomes of the logistic regression analysis are presented in Table 6). Female patients, those aged 75 years or older, those diagnosed with UA, and those with a history of diabetes were more likely to experience delays in seeking medical care. Conversely, patients who attributed their symptoms to heart disease and those who exhibited consultation-seeking behavior at symptom onset were less likely to delay seeking medical help. Multicollinearity diagnostics revealed that all variance inflation factor (VIF) values ranged from 1.333 to 2.766, and tolerance values were all above 0.341, indicating no significant multicollinearity among the predictors.

**Table 5:** Coding scheme of independent variables.

Independent variables	Coding scheme
Sex	Male = 1, Female = 2
Age	60–74 years = 1, ≥75 years = 2
Cohabitation status	Cohabiting = 0, Living alone = 1
Annual household income per capita	Below average = 1, Average or above = 2
Diagnosis	AMI = 1, UA = 2
Perceived disease severity	Mild = 1, Moderate = 2, Severe = 3
Attribute symptoms to cardiac disease	No = 0, Yes = 1
Consultation-seeking behavior	No = 0, Yes = 1
History of heart disease	No = 0, Yes = 1
History of PCI or CABG	No = 0, Yes = 1
History of diabetes	No = 0, Yes = 1

AMI, acute myocardial infarction; UA, unstable angina; PCI, percutaneous coronary intervention; CABG, coronary artery bypass grafting.

**Table 6:** Logistic regression analysis of factors influencing patient delay in older patients with ACS.

Variables	*β*	SE	*p*-value	OR	95% CI of OR	
Sex (Female)	−1.068	0.472	0.024	0.344	0.136	0.867
Age (≥75 years)	−1.632	0.471	0.001	0.196	0.078	0.493
Diagnosis (UA)	−1.333	0.465	0.004	0.264	0.106	0.657
Attribute symptoms to cardiac disease (yes)	1.911	0.487	0.000	6.763	2.606	17.548
Consultation-seeking behavior (yes)	1.657	0.456	0.000	5.244	2.147	12.808
History of heart disease (yes)	1.354	0.521	0.009	3.875	1.395	10.761
History of diabetes (yes)	−1.126	0.481	0.019	0.324	0.126	0.833
Constant	0.415	0.791	0.600	1.514		

UA, unstable angina; SE, standard error; CI, confidence interval; OR, odds ratio.

Variables entered into the initial multivariable model (all with *p* < 0.05 in univariate analysis): sex, age, cohabitation status, annual household income per capita, diagnosis, perceived disease severity, symptoms attributed to cardiac disease, consultation-seeking behavior, history of heart disease, history of PCI/CABG, and history of diabetes (11 variables in total).

Backward stepwise elimination based on the likelihood ratio test was used with a removal criterion of *p* > 0.05. The final model retained 7 variables as shown above.

Variables eliminated during the stepwise process (with *p*-values at the time of elimination): cohabitation status (*p* = 0.460), annual household income per capita (*p* = 0.422), perceived disease severity (*p* = 0.050), and history of PCI/CABG (*p* = 0.220).

Events-per-variable (EPV) ratio in the final model: 67/7 = 9.6.

Multicollinearity diagnostics: all variance inflation factor (VIF) values ranged from 1.333 to 2.766, indicating no significant multicollinearity.

Hosmer–Lemeshow goodness-of-fit test: *χ*^2^ = 1.749, *p* = 0.988.

### 3.5 Predictors mapped to Andersen’s Behavioral Model domains

When mapped to Andersen’s Behavioral Model, the seven independent predictors retained in the final model were distributed across the domains as follows:

(i) Predisposing factors: female sex, age ≥75 years.

(ii) Need factors: diagnosis of UA, history of diabetes, history of heart disease, attributing symptoms to cardiac disease, consultation-seeking behavior.

(iii) Enabling factors: none retained in the final model.

## 4 Discussion

### 4.1 Overview

Excessive pre-hospital delays can postpone the implementation of therapeutic interventions, leading to poorer outcomes in patients with ACS (15, 16). Patient delay is considered the most significant factor contributing to pre-hospital delay (9, 17, 18). In this study of 153 older patients with ACS who survived to hospital admission, the median delay time was 100 min, with 43.8% of patients seeking medical care more than 120 min after symptom onset. The median delay time observed in our hospital-admitted cohort was shorter than that reported in other Chinese studies. Hu et al. (13) investigated 33,386 patients with AMI from multiple centers nationwide using data collected from 2014 to 2019 and reported a median pre-hospital delay of 3.3 h. Peng et al. (19) studied 1,088 patients with STEMI in a single-center study in Beijing using data collected from 2004 to 2007 and found a median delay of 150 min. The shorter delay in our study may be attributable to advancements in healthcare delivery and improved public awareness of ACS symptoms over time, as well as the fact that our study was conducted in a socioeconomically developed region of Eastern China with relatively abundant medical resources. Compared with that in other developing countries (11, 20, 21), the patient delay time in this study was relatively shorter and comparable to the time reported in developed countries (22).

### 4.2 Factors associated with patient delay: mapping to Andersen’s Behavioral Model

The findings of this study provide valuable insights into the factors associated with patient delay in older patients with ACS, organized according to Andersen’s Behavioral Model. When mapped to the model domains, the seven independent predictors retained in the final model were distributed as follows: predisposing factors (sex, age), need factors (diagnosis of UA, history of diabetes, history of heart disease, attributing symptoms to cardiac disease, consultation-seeking behavior), and enabling factors (none retained).

#### 4.2.1 Predisposing factors (non-modifiable risk markers)

Research on the impact of sex on delay in patients with ACS remains inconclusive. Several studies (13, 22–24) have indicated that women with ACS experience longer decision-making times than do men, possibly due to symptom underestimation linked to a lower incidence of ACS and atypical symptoms in women (23, 25). The pain/discomfort scores among women were slightly higher than those among men (26). Additionally, Chinese women’s multiple roles in family, society, and work often lead them to prioritize others’ needs over self-care, contributing to delays in seeking medical care. In contrast, as no significant sex differences exist in ACS symptom presentation patterns (sudden vs. gradual onset) (27), other studies (12, 19, 21) failed to identify sex-specific effects on patient delay in patients with ACS. In this study, advanced age and diabetes were significantly associated with delay among patients with ACS, consistent with previous findings. Older patients generally exhibit longer care-seeking decision intervals than younger patients (3, 12, 28), and this disparity is pronounced in those aged 75 years or older (29). This phenomenon may be related to deterioration in physical function in older patients, leading to atypical symptoms rather than severe chest pain (30). While sex and age are non-modifiable, they help identify high-risk subgroups for targeted education and outreach.

#### 4.2.2 Need factors (distinguishing risk markers from actionable targets)

Among need factors, we identified both risk markers (UA diagnosis, diabetes) and highly actionable targets (history of heart disease, symptom attribution, consultation-seeking behavior).

Diabetes is a significant risk factor for coronary artery disease (CAD), and it is also associated with unrecognized myocardial infarctions and more atypical symptoms (31), which may lead to delayed recognition of symptoms, consequently resulting in patient delay. The findings of this study indicate that older patients with UA experience longer decision-making delays than those with AMI, consistent with the findings of Darawad et al. (11) and DeVon et al. (32). Compared with patients with AMI, those with UA are less likely to experience persistent chest pain and more likely to have chest tightness or intermittent discomfort, leading to different coping strategies for symptoms management. These findings suggest that patients with UA or diabetes require targeted education emphasizing that even mild or atypical symptoms warrant prompt evaluation.

Patients with a history of heart disease are expected to recognize symptoms, attribute them to cardiac disease, and seek treatment promptly (33, 34). Similarly, our study yielded comparable results. Consultation-seeking behaviors following symptom onset and attributing symptoms to cardiac disease were protective factors in this study. Individuals who do not consider themselves at risk for ACS or believe that their current symptoms are caused by ACS may delay seeking medical help (35). An interesting finding was that although patient experience was associated with a reduced decision time for seeking medical care, a history of PCI or CABG was not retained in the multivariate regression model. In China, the widespread implementation of tertiary prevention strategies for CAD has led to earlier detection of coronary blockages in older patients through routine health screenings, prompting proactive treatment before symptom exacerbation. Therefore, older patients with a history of PCI or CABG do not always experience symptom distress owing to ACS, leading to difficulties in symptom recognition. Furthermore, individuals who have undergone revascularization may delay seeking care because of misconceptions that prior interventions preclude disease recurrence.

The protective effects of symptom attribution and consultation-seeking behavior are particularly actionable. These findings highlight the need for targeted ACS education programs to improve symptom awareness in older adults. Specifically, such programs should encourage older adults to actively consult with family members or social networks when experiencing potential cardiac symptoms, as this step can facilitate timely decision-making. For older patients diagnosed with CAD, follow-up visits should be strengthened to enhance patient-doctor interactions, regularly guide and supervise patients in disease management (36), and provide timely counseling during symptom onset. Additionally, professions can involve family members in discharge education, training them to recognize ACS symptoms and provide appropriate advice when consulted.

#### 4.2.3 Enabling factors (contextual considerations)

Although enabling factors such as income, insurance type, and travel time were included in the initial analysis, none were retained in the final multivariable model. This may reflect the relatively resource-rich study setting where access-related barriers are less pronounced. However, these factors may be more influential in rural or resource-constrained settings, warranting further investigation.

### 4.3 Limitations

This study has some limitations. First, the analysis was restricted to patients with ACS from a single tertiary hospital located in a socioeconomically developed provincial capital in Eastern China. The relatively abundant medical resources in this setting-including advanced emergency services, better transport access, and higher health awareness-may explain the shorter median delay time (100 min) compared to some previous domestic studies. Consequently, our findings may not be generalizable to rural or resource-constrained populations. Future multicenter studies across diverse regions and hospital levels are needed to confirm these results and develop tailored interventions. Second, due to the nature of our hospital-based study design, we only included patients who survived to hospital admission and were cognitively able to recall symptom details. This introduces a potential survivorship bias. Consequently, our findings are primarily representative of older ACS patients who reached the hospital alive and may not be generalizable to those who died before receiving medical attention. Third, although data were collected within 24 h of admission to minimize recall bias, the possibility remains that patients’ recollection of symptom onset time may be imprecise, potentially affecting the accuracy of the calculated delay times. Additionally, Andersen’s Behavioral Model allows for broad variable selection; therefore, not all variables can be included. Furthermore, the dichotomization of continuous variables like age and travel time, while aiding clinical interpretability, may have resulted in a loss of statistical power and information. Finally, as a cross-sectional study, this design precludes establishing causal relationships between variables and outcomes.

## 5 Conclusion

This study systematically identified the factors influencing patient delay in older patients with ACS using Andersen’s Behavioral Model. Female sex, advanced age (≥75 years), UA diagnosis, and a history of diabetes were associated with prolonged patient delays. Conversely, attributing symptoms to cardiac disease, seeking medical consultation following symptom onset, and having a history of heart disease were associated with shorter decision times. Therefore, community-based health education campaigns should be prioritized to enhance older adults’ awareness of ACS symptoms, with targeted efforts toward females, older adults aged ≥75 years, individuals with diabetes, and those with cardiovascular high-risk factors but no prior cardiac diagnosis. Simultaneously, structured community health management systems should be established to facilitate direct consultation channels between older residents and healthcare providers, enabling timely decision-making regarding care-seeking behavior when experiencing physical discomfort.

## Data Availability

The raw data supporting the conclusions of this article will be made available by the authors, without undue reservation.
