# Supplementary figures and images for: Influencing factors of patient delay for decision-making in older patients with acute coronary syndrome: a study based on the Andersen’s Behavioral Model
Source: Front Public Health. 2026 Mar 20;14:1788401. doi: 10.3389/fpubh.2026.1788401 (PMC13047189; doi:10.3389/fpubh.2026.1788401)

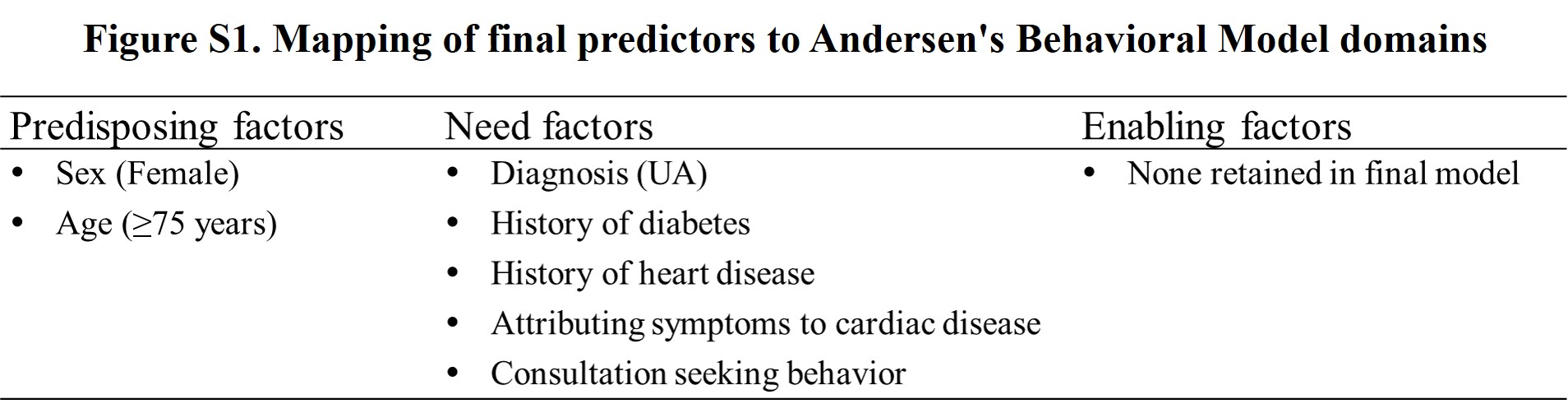

Supplement: Supplementary file 1 [file Image_1.jpeg]
